# Complete Genome Sequence of *Emiliania huxleyi* Virus Strain M1, Isolated from an Induced *E. huxleyi* Bloom in Bergen, Norway

**DOI:** 10.1128/mra.00071-22

**Published:** 2022-04-19

**Authors:** Amir Fromm, Daniella Schatz, Shifra Ben-Dor, Ester Feldmesser, Assaf Vardi

**Affiliations:** a Department of Plant and Environmental Sciences, Weizmann Institute of Science, Rehovot, Israel; b Bioinformatics Unit, Life Sciences Core Facilities, Weizmann Institute of Science, Rehovot, Israel; KU Leuven

## Abstract

*Emiliania huxleyi* virus strain M1 (EhVM1), a large double-stranded DNA virus from the family *Phycodnaviridae*, was isolated from an *Emiliania huxleyi* bloom during a mesocosm experiment in Raunefjorden, Bergen, Norway. Here, we report its complete genome, composed of one full contig.

## ANNOUNCEMENT

*Emiliania huxleyi* is a unicellular alga that forms massive blooms that cover vast oceanic ranges. *E. huxleyi* blooms are routinely infected by the *Emiliania huxleyi* virus (EhV), leading to their demise (1). EhV is a large, double-stranded DNA virus from the family *Phycodnaviridae* (2). Here, we report the complete genome sequence of EhV strain M1 (EhVM1), which was isolated from an *E. huxleyi* bloom during a mesocosm experiment in Bergen, Norway (3, 4). To isolate EhV strains from the natural environment, water from the induced blooms in mesocosm bags (3) was filtered through a GF/C filter and stored at 4°C.

This water was used to inoculate *E. huxleyi* cells and conduct plaque assays for viral isolation, according to the methods described previously for EhV86 (5). Visible plaques were excised on day 4 postinfection and placed in a fresh *E. huxleyi* CCMP374 culture. Once the culture cleared, the lysate was used for two consecutive plaque assay rounds. Cultures (100 mL) of *E. huxleyi* CCMP374 were grown to exponential phase and then infected with EhVM1. Once the culture cleared, the lysate was filtered to eliminate cell debris, and the viruses were concentrated using a 100-kDa Amicon filter. DNA was extracted from the virions by a conventional phenol-chloroform method (6). The DNA concentration and quality were measured using Qubit and NanoDrop analyses. Library preparation was performed according to the Pacific Biosciences (PacBio) microbial multiplexing protocol for one Sequel single-molecule real-time (SMRT) Cell (7). Polymerase reads were demultiplexed to subreads and assigned to EhVM1 using SMRT Link analysis (Table 1). Highly accurate circular consensus sequence (CCS) reads were generated from subreads using SMRT Link with default parameters; all tools were run with default parameters unless otherwise specified.

**TABLE 1:** Sequencing data

Parameter	Finding for EhVM1
No. of polymerase reads	6,520
No. of subreads	5.6E+4
No. of subread bases	2.9E+8
No. of CCS reads	2,995

Three draft EhVM1 assemblies were constructed by (i) SMRT Link microbial assembly using an expected genome size parameter of 1 Mbp, (ii) Canu assembly (8) with both CCS reads and subreads as the input, and (iii) SPAdes assembly (9) with CCS reads as the input. A final version of the genome was assembled from the three draft assemblies using GFinisher (10). Circularization of the genome was confirmed with CCS reads that spanned both ends. The GC content of the genome was calculated via an in-house script. The genome sequence of EhVM1 consists of one circular contig of 411,976 bp, longer than the EhV86 genome (407 kbp [5]), with an average GC content of 40.32%. Coding DNA sequences (CDSs) longer than 100 amino acids were predicted using GeneMarkS (11) with the virus sequence type parameter. Additional CDSs were predicted using Prodigal (12). tRNAs were predicted with tRNAscan-SE v2.0 (13) and were analyzed by the RNAcentral (14) web server for verification. We predict that the EhVM1 genome contains 489 CDSs, more than EhV86 (472 CDSs [5]), and 6 tRNA genes.

### Data availability.

The complete genome sequence of EhVM1 has been deposited in GenBank under the accession number OM339720. PacBio sequence reads have been deposited in the NCBI Sequence Read Archive (SRA) under the BioSample accession number SAMN24818830. The complete record is available in the NCBI BioProject database under the accession number PRJNA796183.
